# Comparative Safety and Efficacy of Roux-en-Y Gastric Bypass Versus One-Anastomosis Gastric Bypass: A Systematic Review and Meta-Analysis of Randomized Clinical Trials

**DOI:** 10.7759/cureus.71193

**Published:** 2024-10-10

**Authors:** Mohamed Elsaigh, Bakhtawar Awan, Mohamed Marzouk, Mohamed H Khater, Ahmad Asqalan, Justyna Szul, Doaa Mansour, Nusratun Naim, Omnia S Saleh, Prashant Jain

**Affiliations:** 1 General and Emergency Surgery, Royal Cornwall Hospital, Truro, GBR; 2 General and Emergency Surgery, Northwick Park Hospital, London, GBR; 3 General and Emergency Surgery, Northwick Park Hospital, London North West University, London, GBR; 4 General Surgery, Northwick Park Hospital, London, GBR; 5 Upper GI Surgery, Cairo University Hospitals, Cairo, EGY; 6 General Surgery, Hull University Teaching Hospitals, Hull, GBR; 7 Surgery, Laboratory for Surgical and Metabolic Research, Brigham and Women’s Hospital, Harvard Medical School, Boston, USA

**Keywords:** bariatric surgery, obesity, one-anastomosis gastric bypass, roux-en-y gastric bypass, systematic review and meta analysis

## Abstract

Obesity has become a global epidemic, affecting both developed and developing nations. Despite extensive efforts, historical outcomes of medical interventions for obesity have been unsatisfactory. Bariatric surgeries, including sleeve gastrectomy (SG) and laparoscopic Roux-en-Y gastric bypass (RYGB), are now recognized as the primary treatment for severe obesity. However, laparoscopic one-anastomosis gastric bypass (OAGB) has emerged as a promising alternative, offering simplified procedures compared to RYGB. While OAGB's initial outcomes are optimistic, concerns about biliary reflux persist. Our systematic review aims to compare the safety and efficacy outcomes of RYGB and OAGB to inform clinical decision-making in managing obesity. We searched five databases up to February 2024. We included randomized controlled trials (RCTs) comparing RYGB and OAGB in obese patients, focusing on safety and efficacy outcomes. Data extraction covered study details, participant demographics, interventions, and outcomes related to operative details, complications, follow-up results, and weight changes. The risk of bias was assessed using the Cochrane tool. The analysis involved risk ratios for dichotomous data and mean differences for continuous data, using fixed or random effects models based on heterogeneity. Analyses were performed with Review Manager software v5.4. A total of 1057 patients were included in the analysis, sourced from 12 distinct RCTs. The analysis indicated OAGB outperformed RYGB in BMI reduction (MD = -0.69, p = 0.005), whereas RYGB was more effective in excess weight loss (MD = 6.51, p < 0.0001) and excess BMI loss (MD = 3.91, p < 0.0001). OAGB led to shorter operation times (MD = -34.89 minutes, p < 0.0001) and shorter periods of hospital stays (MD = -0.27 days, p = 0.01), along with fewer overall complications (RR = 0.58, p = 0.02) and lower incidence of upper gastrointestinal endoscopy complications (RR = 2.98, p = 0.0001). On the other hand, RYGB showed higher remission rates for dyslipidemia (RR = 0.60, p = 0.0003) and higher remissions of hypertension (RR = 0.83, p = 0.04). The majority of results were homogenous. Both OAGB and RYGB have their respective advantages and limitations. OAGB appears to offer benefits in terms of operation efficiency and early postoperative recovery, making it a potentially preferable option for patients and surgeons focused on these aspects. On the other hand, RYGB might be more suitable for patients prioritizing long-term weight loss and remission of certain comorbidities like hypertension. Ultimately, the choice between OAGB and RYGB should be made on an individual basis, considering the specific needs, conditions, and goals of each patient.

## Introduction and background

The escalating prevalence of obesity observed both in developing and developed nations, is characterized as a worldwide pandemic [1]. In 2016, 39% of adults aged 18 years and above were classified as overweight, with 13% categorized as obese. The world is seeking the best interventions to increase the quality of life for the overweight and obese populations [2]. Dietary recommendations, lifestyle adjustments, exercise, behavioral therapy, and adjunctive pharmacotherapy with medications like sibutramine and orlistat have demonstrated limited effectiveness [3]. The historical outcomes of medical interventions for obesity have been underwhelming. As indicated by the National Institutes of Health Consensus Conference in 1991 [4], a definitive surgical solution for obesity remained elusive until its discovery. Bariatric surgeries are the medical procedures that are conducted on individuals who are experiencing severe overweight or obesity [5]. Bariatric surgery is alternatively termed as weight loss surgery. These surgical interventions are designed to facilitate weight reduction by modifying the anatomical structure of the digestive system [6]. The alterations of the anatomical structure of the digestive system would lead to a reduction of food intake, a decrease in nutrient absorption, or both at the same time [6, 7]. At present, bariatric surgery stands as the singular effective approach yielding enduring weight loss and alleviating obesity-associated comorbidities [8].

Sleeve gastrectomy (SG) stands as the most commonly embraced procedure although it may carry postoperative morbidity [9]. Laparoscopic Roux-en-Y Gastric Bypass (RYGB) is considered the most preferred management for morbid obesity, demonstrating sustained and substantial weight loss over the long term while also ameliorating obesity-related comorbidities [10]. One-anastomosis gastric bypass (OAGB) presents various evident benefits compared to RYGB, such as a singular anastomosis, reduced internal defects for herniation, and simplified construction and revision procedures. Nonetheless, apprehensions regarding symptomatic biliary reflux and its potential adverse effects on the stomach and esophagus have hindered its widespread acceptance [11]. Both techniques have gone through evolutions as numerous technical aspects of RYGB have experienced modifications, encompassing the creation of the gastric pouch, the method of anastomosis using staplers or hand-sewn techniques, and adjustments in limb lengths [12]. While some individuals express optimism regarding the promising initial outcomes of OAGB [13], there exist reservations among others regarding the potential development of adverse biliary reflux and its enduring ramifications due to a gastrojejunal anastomosis similar to that observed in Billroth II procedures [14].

OAGB is a less frequently utilized method compared to RYGB although OAGB is increasing in popularity due to being a simplified procedure compared to RYGB [15-17]; so in our systematic review and meta-analysis, we are aiming to investigate the differences between the two techniques considering the safety and efficacy outcomes.

## Review

Methods

Our research was structured in accordance with the Cochrane Handbook for Systematic Review of Interventions and followed the Preferred Reporting Items for Systematic Reviews and Meta-analyses (PRISMA) guidelines for reporting [18, 19].

Eligibility Criteria and Study Selection

Two reviewers independently evaluated the retrieved references based on predetermined eligibility criteria. Inclusion criteria encompassed trials involving patients that went through RYGB or OAGB whether the patients were diabetic or obese. Any evaluated safety and efficacy outcomes. Additionally, the included studies have to be a randomized controlled trial (RCT) design. Conversely, exclusion criteria involved letters, cohorts, animal studies, non-randomized trials, non-English publications, abstract-only publications, and studies with unreliable data for extraction and analysis.

Quality and Risk of Bias (ROB)

We evaluated the quality of the included trials using the Cochrane risk of bias assessment tool designed for RCTs [20]. This tool assesses a range of factors, encompassing selection, performance, detection, attrition, reporting, and other potential sources of bias. Assessments were categorized as "high", "low", or "unclear" risk of bias. Any discrepancies were resolved through discussion between the reviewers or by involving a third assessor.

*Dat* *a Extraction*

Data extraction was conducted utilizing an offline data extraction sheet, with careful verification to prevent duplication of published data. The following information was extracted as baseline and summary: study ID, intervention type, country, age, gender (male), preoperative BMI, hypertension, metabolic syndrome, diabetes, follow-up, primary outcome, and inclusion criteria. Our outcomes were divided into three main points as follows: 1- Post-operative outcomes, 2- follow-up, and 3- Weight. The postoperative outcomes were: 1- operation time, 2- hospital stay, 3- intraoperative complications, 4- early postoperative complications, and 5- upper gastrointestinal endoscopy complications. The follow-up outcomes were: 1- Gastroesophageal reflux disease (GERD) symptoms, 2- Dumping syndrome, 3- Anemia, 4- Osteoarthritis (remission), 5- Dyslipidemia (remission), 6- Hypertension (remission), 7- T2DM (remission). And finally, the weight outcomes were: 1- BMI, 2- Total weight loss, 3- Excess weight loss, and 4- 2-Excess BMI loss.

Analysis

For dichotomous outcomes, our analysis involved synthesizing risk ratios (RR) with corresponding 95% confidence intervals (CIs). Continuous outcomes were represented as mean differences (MDs), also accompanied by 95% CIs. Initially, we applied a fixed effects model when the effect estimate stemmed from homogeneous studies; otherwise, a random effects model was utilized. Heterogeneity among studies was assessed using the I2 statistic and chi-squared test, with p-values exceeding 0.1 indicating heterogeneity, and I2 values of 50% or greater suggesting significant heterogeneity. Additionally, statistical analyses were performed utilizing Review Manager software version 5.4.

Results

Literature Search and Study Selection

**Figure 1:**
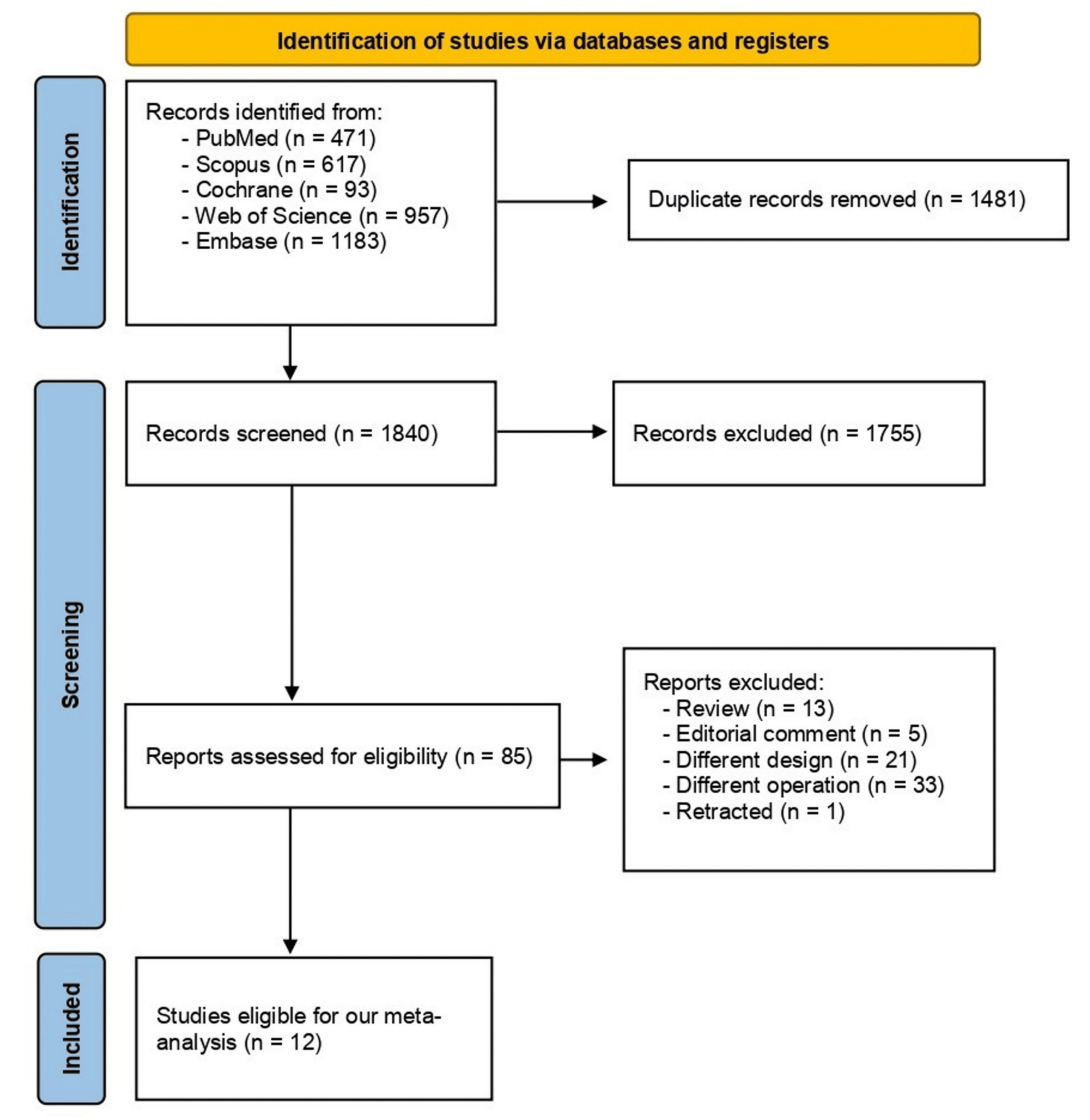
PRISMA Flow diagram PRISMA: Preferred Reporting Items for Systematic Reviews and Meta-Analyses

Our search using five databases found 3321 studies. After duplicate removal which resulted in 1481 papers being eliminated, 1840 studies were qualified for screening. After title and abstract screening, we excluded 1755 papers, and 85 articles entered full-text screening. We excluded 13 reviews, five editorial studies, 21 papers of different study designs, 33 studies with different operations, and one retracted paper. Eventually, 12 articles met our criteria and were finally included in our systematic review and analysis [14, 21-31]. All the data are shown in Figure 1.

Study Characteristics

Our systematic review and meta-analysis consisted of 12 RCTS with a total population of 1057 patients. The number of populations in the two interventions was similar as the allocation of the population in the included RCTs was equal. The included studies were multicentered as it was made in several countries such as Egypt, Netherlands, Finland, Brazil, China, Venezuela, France, and India. The mean age of the included population ranged from 35 years to 45 years in most of the included studies. The vast majority of the included population consisted of females in comparison to males. All the preoperative BMI indicated obesity with a higher number than 45 BMI. The incidence of hypertension, metabolic syndrome, and diabetes was various in the included studies with significant numbers of patients suffering from these morbidities. The follow-up periods in the included studies were 6, 12, and 24 months in all of the studies except for Singh et al. which had extended follow-up periods of up to four years. The baseline and summary characteristics of all the included RCTs are presented in Table 1.

**Table 1:** Baseline and summary. T2D: Type 2 diabetes; T2DM: Type 2 diabetes mellitus; RYGB: Roux-en-Y gastric bypass; SR: Systematic review; BMI: Body mass index; GERD: Gastroesophageal reflux disease; EBWL: Excess body weight loss

Study ID	Intervention (n)	Country	Age(year)	Gender(male)	Preoperative BMI	Hypertension	Metabolic syndrome	Diabetes	Follow-up	Primary outcome	Inclusion criteria
Abd El-Sadek et al., 2021 [31]	RYGB (n=50)	Egypt	39.20 ± 6.76	9 (18%)	56.73 ± 4.69	25 (50%)	25 (50%)	22 (44%)	12, 24 months	The weight loss in terms of EWL% and excess BMI loss percent (EBMI loss%), and remission/improvement of comorbidities.	All the patients included during the period from September 2018 to December 2020, 100 patients with super obesity. Included patients from both genders, aged 18-60 years with super obesity (BMI ≥ 50 kg/m^2^) with or without comorbidities.
	OAGB (n=50)		40.50 ± 6.67	12 (24%)	59.70 ± 4.32	21 (42%)	28 (56%)	21 (42%)			
Eskandaros et al., 2021 [21]	RYGB (n=40)	Egypt	36 ± 11	20 (50%)	50.01 ± 3.50	18 (45%)	NA	13 (32.5%)	6, 12 months	The weight loss in terms of EWL% and excess BMI loss percent (EBMI loss%), and remission/improvement of comorbidities.	The paper had 80 patients (out of 457 screened) with mild-to-moderate GERD who were recruited from the bariatric outpatient clinic from January 2017 until December 2020 and consented to maintain the follow-up schedule and to repeat the investigations at 6 and 12 months.
	OAGB (n=40)		36 ± 10	19 (47.5%)	49.78 ± 3.40	14 (35%)	NA	15 (37.5%)			
Fahmy et al., 2018 [22]	OAGB (n=30)	Egypt	31.3 ± 8.0	3 (10%)	45.5 ± 5.3	6 (20.0%)	NA	9 (30.0%)	12 months	The postoperative GERD score	Any patient with a body mass index (BMI) >40 kg/m^2^ or BMI >35 kg/m^2^ with comorbidities (hypertension, diabetes mellitus, and osteoarthritis) with documented failure of weight-loss attempts for at least 6 months and good motivation for surgery. The age was restricted to patients from 18 to 59 years old.
	RYGB (n=30)		32.7 ±7.3	3 (10%)	44.1 ± 4.7	4 (13.3%)	NA	8 (26.6%)			
Hany et al., 2022 [23]	OAGB (n=80)	Netherlands	42.6±7.1	11 (13.7%)	45.1 ± 8.3	11 (13.8%)	NA	12 (15.0%)	6, 12, 24 months	The weight loss, the occurrence of complications, nutritional laboratory test results, and resolution or improvement of associated medical problems after the weight loss.	Any patient aged between 18 and 60 years. Weight regain was the main inclusion criterion, defined as any increase in weight above the nadir as reported by the patient. The mean BMI at the time of revisional surgery was around 45 kg/m^2^.
	RYGB (n=80)		43.4 ± 7.5	11 (13.7%)	44.9 ± 6.6	14 (17.5%)	NA	6 (7.5%)			
Heinonen et al., 2023 [24]	RYGB (n=59)	Finland	47.1 ± 1.05	14 (23.72%)	43.8 ± 0.78	42 (71.2%)	NA	26 (44.8%)	6, 12 months	The difference in percent excess weight loss at one year	Patients with age >18 years, BMI ≥35 kg/m^2^, eligible for gastric bypass surgery according to national treatment guidelines, and Willingness to participate in this trial
	OAGB (n=60)		46.6 ± 1.03	19 (3.67%)	44.6 ± 0.78	36 (60%)	NA	27(45%)			
Ibrahim et al., 2022 [25]	OAGB (n=30)	Egypt	37.7 ± 9.9	4 (13%)	53.5 ± 9.4	7 (23.3%)	NA	17 (56.7%)	12 months	The weight loss	Any morbidly obese patients 18 to 60 years old with acceptable operative risks. All patients should have failed an adequate conservative program for at least 6 months and were able to comply with nutritional supplementation and long-term follow-up. Morbid obesity was defined as a BMI>40 kg/m^2^ or >35 kg/m^2^ with obesity-related comorbidities.
	RYGB (n=35)		36.9 ± 10.2	14 (40%)	52.3 ± 5.1	11 (31.4%)	NA	22 (62.9%)			
Katayama et al., 2021 [26]	OAGB (n=10)	Brazil	39.5 ± 7.0	0 (0%)	43.2 ± 3.7	4 (40%)	NA	1 (10%)	6 months	The weight loss and other related outcomes.	Any patients with age between 20–60 years, and body mass index (BMI) between 35 kg/m² and 50 kg/m².
	RYGB (n=10)		42.2 ± 9.3	1 (10%)	43.1 ± 3.9	7 (70%)	NA	5 (50%)			
Lee et al., 2005 [27]	OAGB (n=40)	China	30.7 ± 8.4	13(32.5%)	44.8 ± 8.8	NA	22 (55%)	NA	12-24 months	The total weight loss and quality of life	Patients with a history of obesity of 5 years’ duration; BMI 40 kg/m^2^ or BMI 35 kg/m^2^ with comorbidities; documented weight loss attempts in the past; and good motivation for surgery. The age was restricted to patients from 18 to 59 years of age.
	RYGB (n=40)		31.1 ± 9.1	12(30%)	43.8 ± 4.8	NA	23 (57.5%)	NA			
Level et al., 2020 [28]	OAGB (n=9)	Venezuela	37.5 ± 6.6	0 (0%)	42.9 ± 5.5	4 (44.44%)	NA	1 (11.1%)	5 years	The weight loss	Patients with age > 18 years, body mass index (BMI) > 40 kg/m^2^, BMI ≥ 35 kg/m^2^ with one or more comorbidities, agree to participate in the study and have signed an informed consent.
	RYGB (n=19)		36.8 ± 9.3	0 (0%)	42.6 ± 5.9	6 (31.57%)	NA	2 (10.5%)			
Robert et al., 2019 [14]	OAGB (n=117)	France	44.4 ± 11.4	37 (32%)	43.8 ± 6.1	38 (33%)	NA	28 (26%)	24 months	The weight loss	Patients with a BMI of 40 kg/m² or higher, or 35 kg/m² or higher with the presence of at least one comorbidity (type 2 diabetes, high blood pressure, obstructive sleep apnoea, dyslipidemia comparison, or arthritis), and aged 18–65 years
	RYGB (n=117)		42.6 ± 10.2	30 (26%)	43.9 ± 5.1	33 (28%)	NA	30 (29%)			
Salman et al., 2023 [29]	OAGB (n=31)	Egypt	36.45 ± 8.70	9 (29%)	48.80 ± 7.83	11 (35.5%)	NA	13 (41.9%)	6 months	The difference between the studied groups in the EBWL	Adults with a BMI of ≥ 40 kg/m^2^ or ≥ 35 kg/m^2^ were suitable candidates for bariatric surgery, provided they tried nonsurgical treatment without success for at least 6 months. Patients who were candidates for RYGB or OAGB, and accepted to participate were included.
	RYGB (n=31)		35.68 ± 9.97	15 (48.4%)	49.29 ± 6.82	9 (29%)	NA	22 (71%)			
Singh et al., 2023 [30]	OAGB (n=25)	India	45.8±9.1	7 (28%)	47.0 ± 6.7	NA	NA	NA	6,12,24,36,48 months	The comparison of the complete remission and improvement rates of type 2 DM following OAGB and RYGB at 6 months, 12 months, and yearly.	All patients with a BMI ≥30 kg/m^2^ with concomitant T2DM were included from October 2017 until December 2021
	RYGB (n=24)		46.6±8.2	8 (33.33%)	44.7 ± 4.9	NA	NA	NA			

Quality of the Included Studies

**Figure 2:**
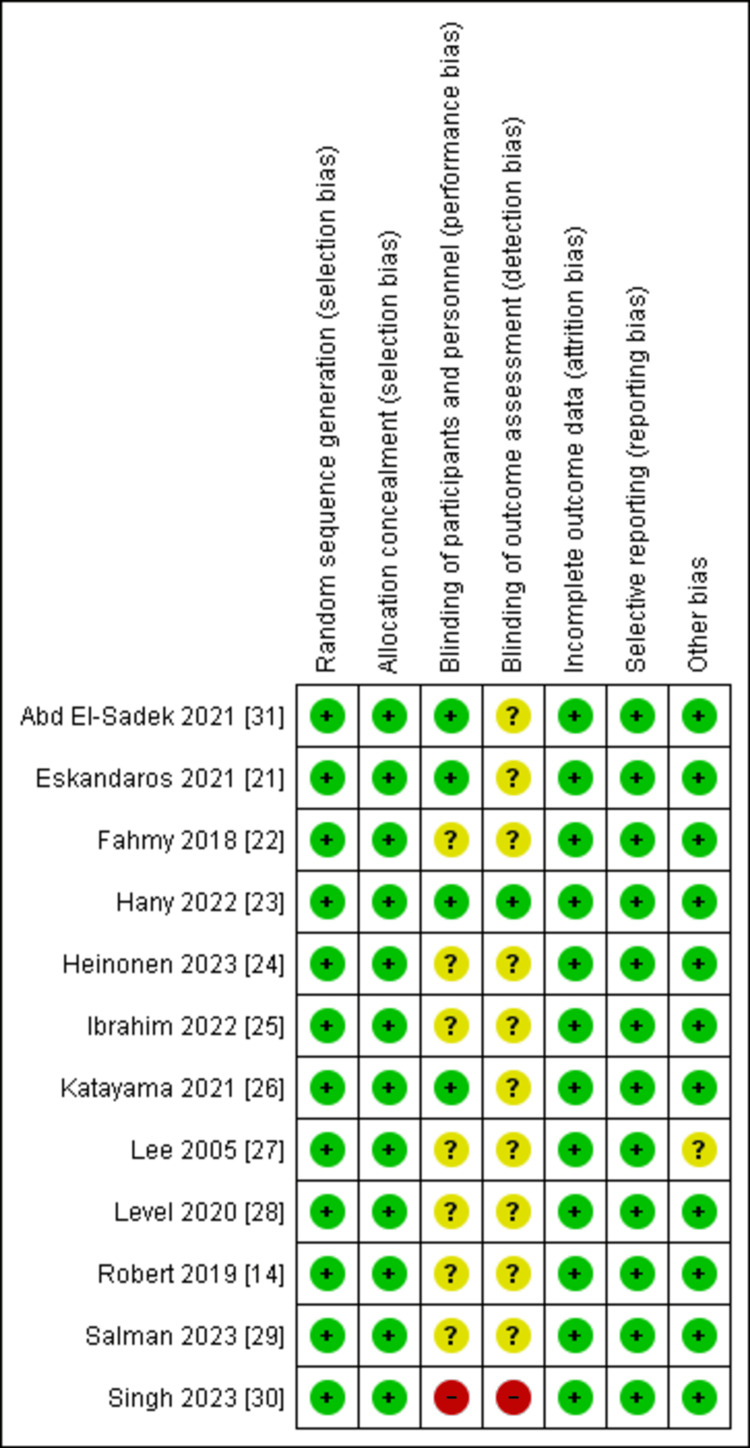
ROB summary. ROB: Risk of bias

Our included studies ranged from high-quality to moderate-quality studies. All of the included studies were associated with a low risk of bias regarding random sequence generation, allocation, and selective or incomplete reporting. The blinding of the participants and personnel responsible for the operation was the main point that resulted in bias in all the included papers. The overall quality of the included papers is considered high with only one study with an unclear risk of bias in the other bias section. A graph showing the ROB is presented in Figure 2.

Postoperative outcomes

Operation Time in Minutes

**Figure 3:**
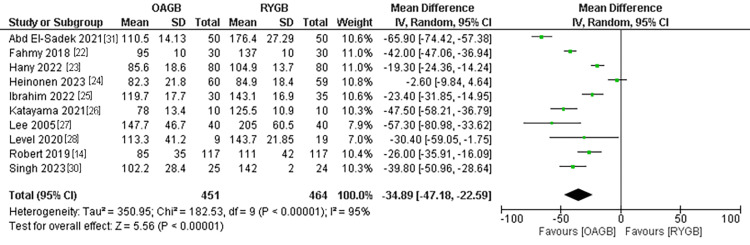
Operative time in minutes

The operation time outcome was estimated in ten different studies with a population of 915 patients. The operation time was significantly shorter in the OAGB technique compared to the RYGB technique. The pooled MD of the studies in minutes was (MD = -34.89, 95% CI = [-47.18, -22.59], p < 0.0001). The pooled results were heterogeneous and couldn’t be fixed with any analytical methods (P<0.00001, I2 =95%) (Figure 3).

Hospital Stay in Days

**Figure 4:**
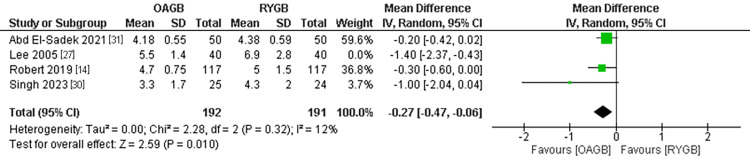
Hospital stay

The hospital stay outcome was estimated in four different studies with a population of 383 patients after excluding Lee et al. (2005) with the leave-one-out method. The hospital stay time was significantly shorter in the OAGB technique compared to the RYGB technique. The pooled MD of the studies in days was (MD = -0.27, 95% CI = [-0.47, -0.06], p = 0.01). The pooled results were homogenous and the heterogeneity was resolved by leaving-one-out method and the results are (P=0.32, I2=12%) (Figure 4).

Intraoperative Complications

**Figure 5:**
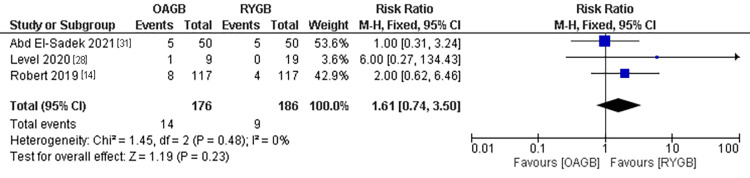
Intraoperative complications

The intraoperative complications outcome was estimated in three different studies with a population of 362 patients. We found no difference between the two techniques although the RYGB technique was associated with a lower incidence of intraoperative complications compared to the OAGB technique. The pooled results were (RR=1.61, 95% CI = [0.74, 3.5], p = 0.23). The pooled results were homogenous (P=0.32, I2 = 12%) (Figure 5).

Early Postoperative Complications

**Figure 6:**
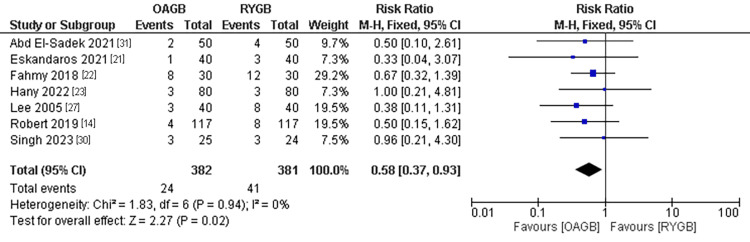
Early postoperative complications

The early postoperative complications outcome was estimated in seven studies with a population of 763 patients. We found a significantly lower incidence of complications in the OAGB technique compared to the RYGB technique. The pooled results were (RR=00.58, 95% CI = [0.37, 0.93], p = 0.02). The pooled results were homogenous (P=0.94, I2=0%) (Figure 6).

Upper Gastrointestinal Endoscopy Complications

**Figure 7:**
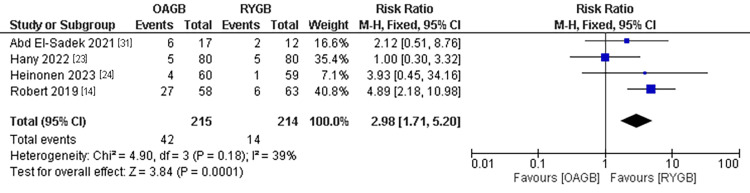
Upper gastrointestinal endoscopy complications

The upper gastrointestinal endoscopy complications outcome was estimated in four studies with a population of 429 patients. We found a significantly lower incidence of complications in the OAGB technique compared to the RYGB technique. The pooled results were (RR=2.98, 95% CI = [1.71, 5.20], p = 0.0001) (Figure 7).

Follow-up outcomes

GERD Symptoms

**Figure 8:**
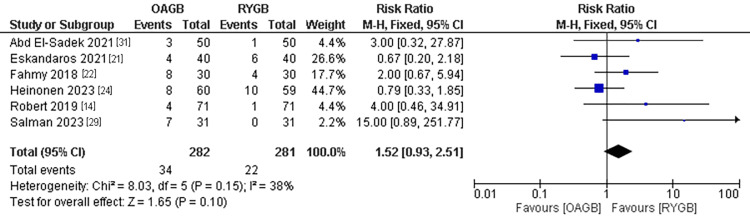
GERD symptoms GERD: Gastroesophageal reflux disease

The GERD symptoms outcome was estimated in six studies with a population of 563 patients. No significant incidence of GERD in the OAGB technique compared to the RYGB technique. The pooled results were (RR=1.52, 95% CI = [0.93, 2.51], p = 0.1). The pooled results were homogenous (P=0.15, I2 =38%) (Figure 8).

Dumping Syndrome

**Figure 9:**
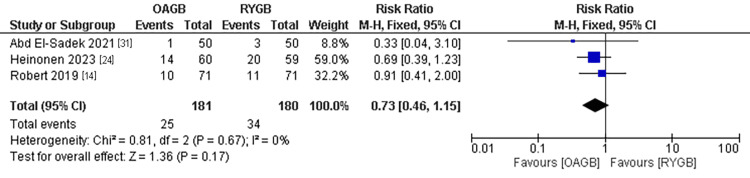
Dumping symptoms

The dumping syndrome was mentioned in three studies with a population of 361 patients. No significant incidence of dumping syndrome was found in the OAGB technique compared to the RYGB technique. The pooled results were (RR=0.73, 95% CI = [0.46, 1.15], p = 0.17). The pooled results were homogenous (P=0.67, I2=0%) (Figure 9).

Anemia

**Figure 10:**
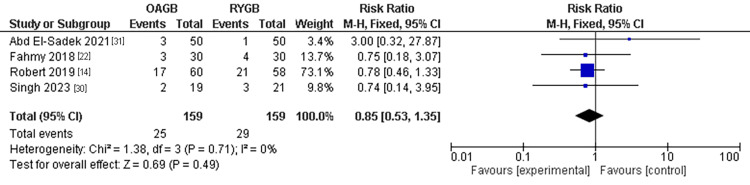
Anemia

The anemia was mentioned in three studies with a population of 361 patients. No significant incidence of anemia was found in the OAGB technique compared to the RYGB technique. The pooled results were (RR=0.85, 95% CI = [0.53, 1.35], p = 0.17). The pooled results were homogenous (P=0.67, I2 =0%) (Figure 10).

Remission of Osteoarthritis

**Figure 11:**
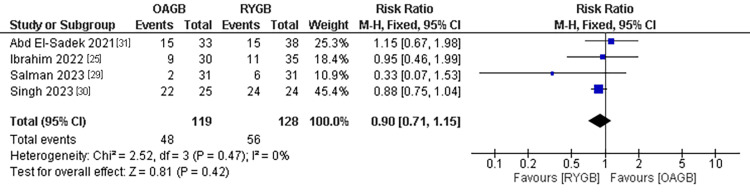
Osteoarthritis (remission)

The osteoarthritis remission was mentioned in four studies with a population of 247 patients. No significant remission of osteoarthritis was found in the OAGB technique compared to the RYGB technique. The pooled results were (RR=0.81, 95% CI = [0.71, 1.15], p = 0.42). The pooled results were homogenous (P=0.47, I2=0%) (Figure 11).

Remission of Dyslipidemia

**Figure 12:**
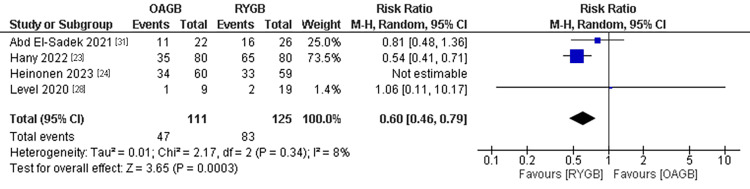
Remission of dyslipidemia

The dyslipidemia remission was mentioned in four studies with a population of 236 patients after excluding Heinonen et al. (2023) with the leave-one-out method. A significantly higher remission rate of dyslipidemia was found in the RYGB technique compared to the OAGB technique. The pooled results were (RR=0.60, 95% CI = [0.46, 0.79], p = 0.0003). The pooled results were homogenous after the leaving-one-out method (P=0.34, I2=8%) (Figure 12).

Remission of Hypertension

**Figure 13:**
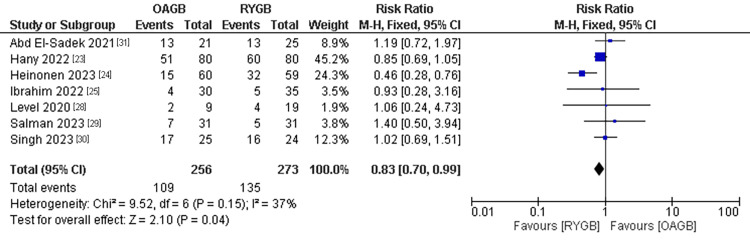
Hypertension (remission)

The hypertension remission was mentioned in seven studies with a population of 529 patients. A significantly higher remission rate of hypertension was found in the RYGB technique compared to the OAGB technique. The pooled results were (RR=0.83, 95% CI = [0.70, 0.99], p = 0.04). The pooled results were homogenous (P=0.15, I2=37%) (Figure 13).

Remission of T2DM

**Figure 14:**
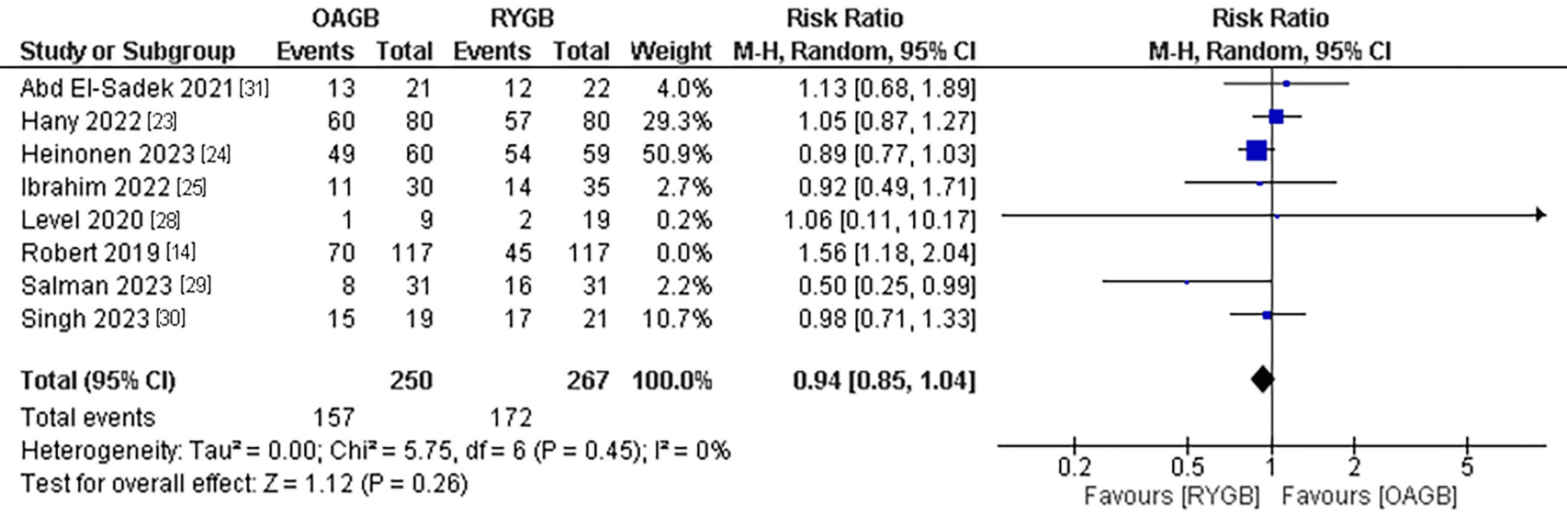
Remission of T2DM

Diabetes remission was mentioned in eight studies with a total population of 517 patients. No significant difference regarding T2DM was found between the two interventions. The pooled results were (RR=0.94, 95% CI = [0.85, 1.04], p = 0.26). The pooled results were homogenous after the use of leaving-one-out method and excluding Robert et al. (2019) (P=0.45, I2 =0%) (Figure 14).

Weight-related outcomes

BMI Change

**Figure 15:**
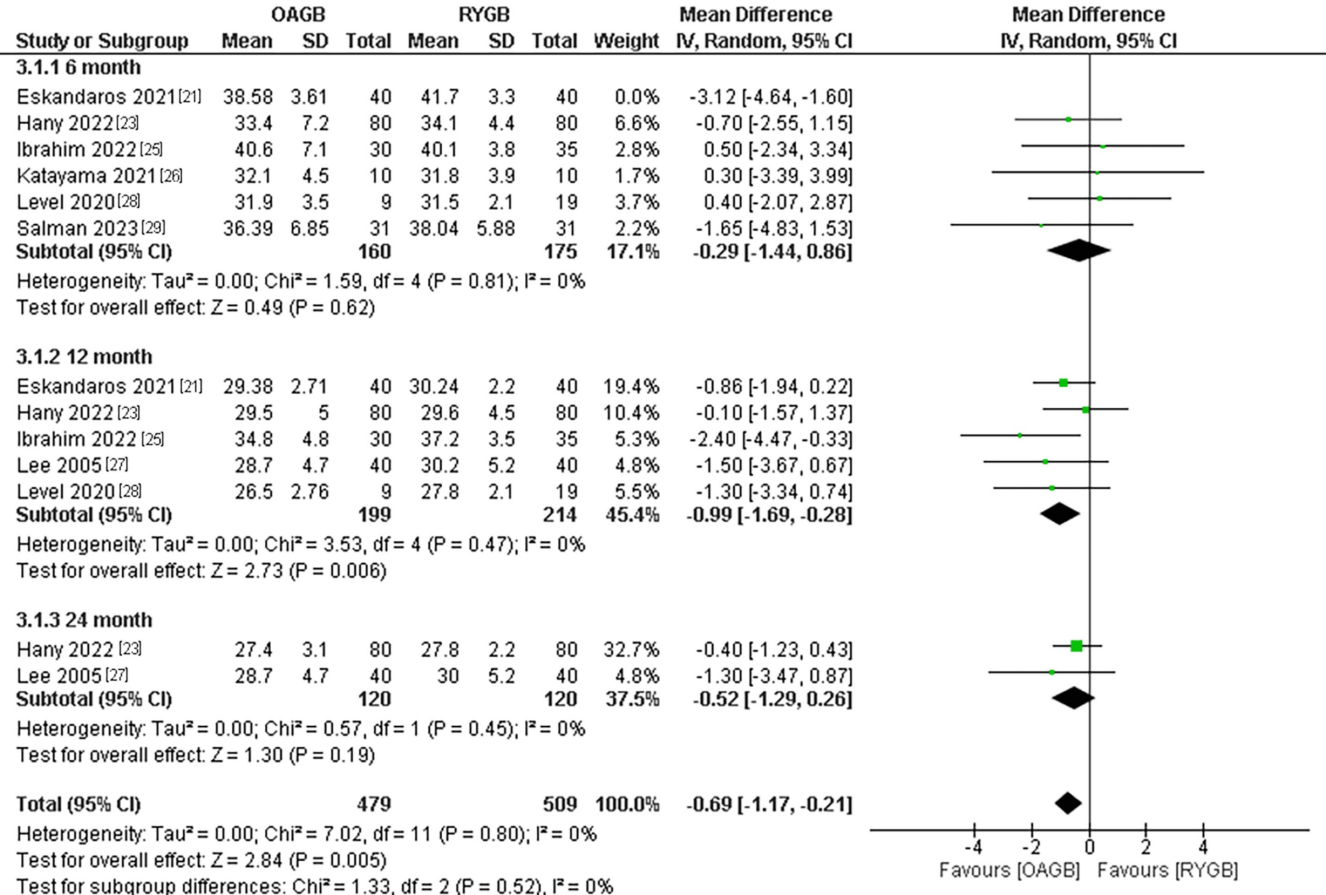
BMI change

The outcome was divided into three subgroups according to the follow-up period. The number of participants in the outcome was 988. There was no significant difference between the two interventions in the six-month follow-up time and the 24-month follow-up time; and the pooled results were (MD= -0.29, 95% CI = [-1.44, 0.86], p = 0.62), and (MD = -0.52, 95% CI = [-1.29, 0.26], p = 0.19), respectively. On the other hand, the 12-month outcomes showed a significant difference regarding the change in BMI. The OAGB was found to have a significantly higher change in BMI compared to RYGB, and the pooled results were (MD = -0.99, 95% CI = [-1.69, -0.28], p = 0.006). Additionally, the total combined results showed a significant superiority of OAGB when compared to RYGB regarding BMI change and the results were (MD = -0.69, 95% CI = [-1.17, -0.21], p = 0.005). All the results were homogenous and we used the leave-one-out method to exclude the results of Eskandaros et al. (2021) in the six-month follow-up period (overall p-value=0.80, I2=0%) (Figure 15).

Total Weight Loss

**Figure 16:**
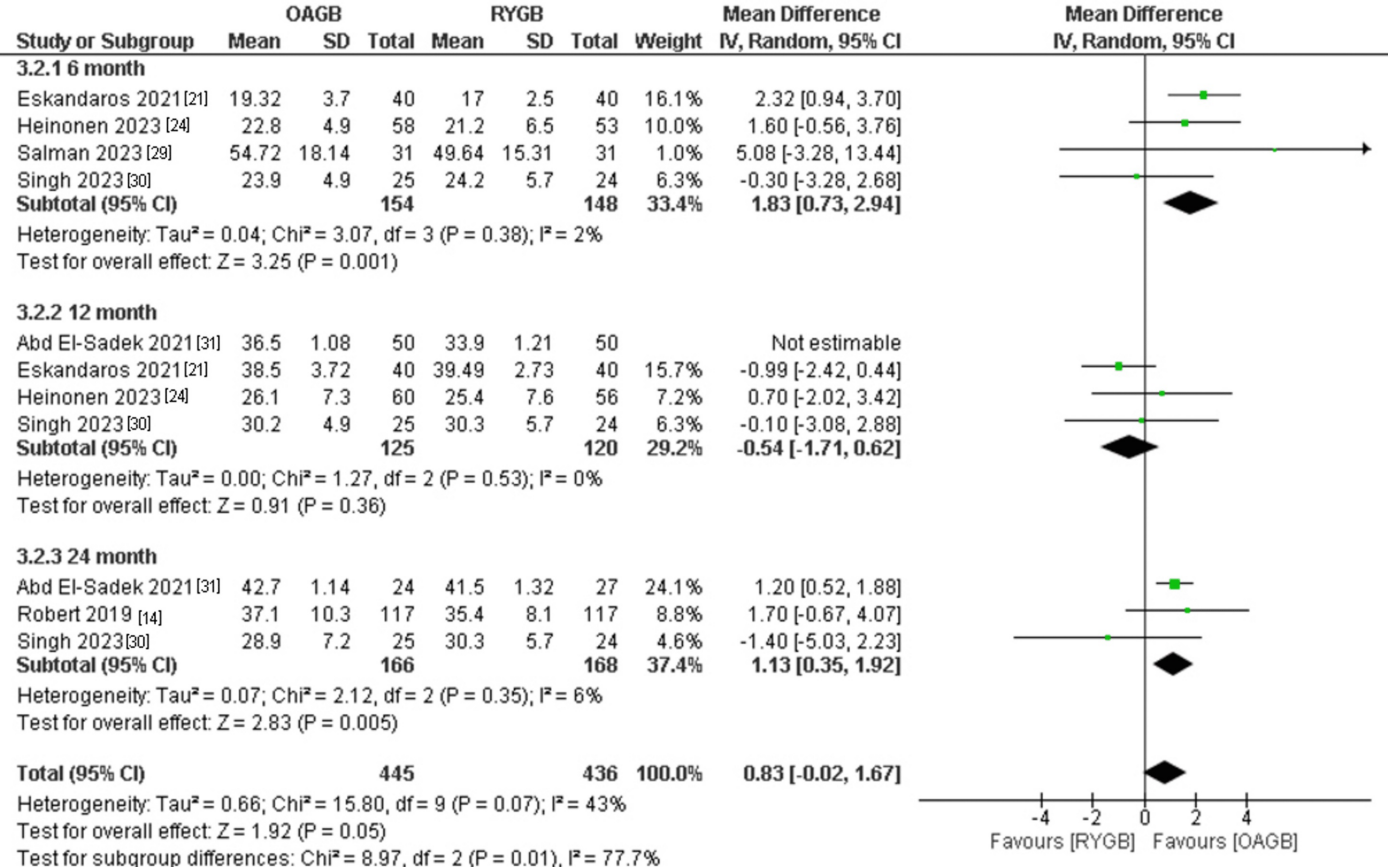
Total weight loss

The outcome was divided into three subgroups according to the follow-up period. The number of participants in the outcome was 881. There was no significant difference between the two interventions in the 12-month follow-up time (MD = -0.54, 95% CI = [-1.71, 0.62], p = 0.36). In the six months follow-up time, RYBG showed a significantly higher total weight loss and the results were (MD = 1.83, 95% CI = [0.73, 2.94], p = 0.001). In the 24-month follow-up time, RYBG showed a significantly higher total weight loss and the results were (MD = 1.13, 95% CI = [0.35, 1.92], p = 0.005). The total combined results of all the follow-up periods showed no significant superiority of RYGB when compared to OAGB regarding total weight loss and the results were (MD = 0.83, 95% CI = [-0.02, 1.67], p = 0.05). All the results were homogenous after the use of the leave-one-out method to exclude the results of Abd El-Sadek et al. (2021) in the 12-month follow-up period (overall p-value=0.07, I2=43%) (Figure 16).

Excess Weight Loss

**Figure 17:**
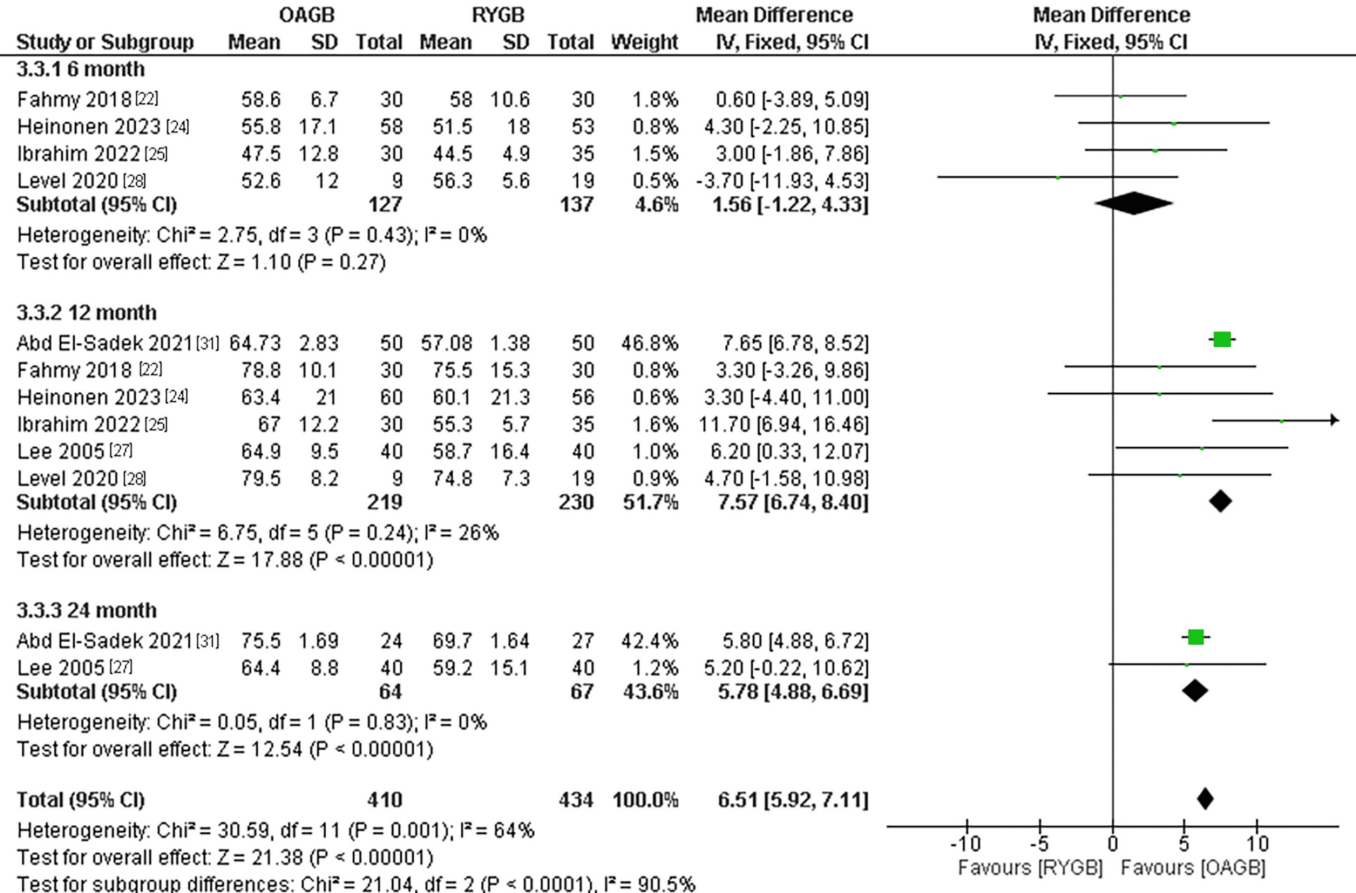
Excess weight loss

The outcome was divided into three subgroups according to the follow-up period. The number of participants in the outcome were 844. There was no significant difference between the two interventions in the six-month follow-up time and pooled results were (MD = 1.56, 95% CI = [-1.22, 4.33], p = 0.27). In the 12-month follow-up time, RYBG showed a significantly higher excess weight loss and the results were (MD = 7.57, 95% CI = [6.74, 8.40], p < 0.0001). Similar results were found in the 24-month follow-up time, as RYBG showed a significantly higher excess weight loss and results were (MD = 5.78, 95% CI = [4.88, 6.69], p < 0.0001). The total combined results of all the follow-up periods showed a significant superiority of RYGB when compared to OAGB regarding excess weight loss and the results were (MD = 6.51, 95% CI = [5.92, 7.11], p <0.0001). The overall results were heterogeneous (overall p value=0.001, I2=64%) (Figure 17).

Excess BMI Loss

**Figure 18:**
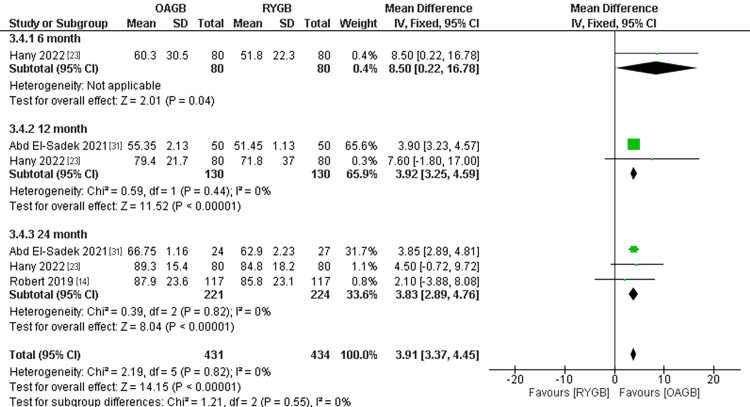
Excess BMI loss

The outcome was divided into three subgroups according to the follow-up period. The total number of participants in the outcome was 865. All the subgroup results showed that RYGB was superior regarding excess BMI loss. In the 12-month follow-up time, RYBG showed a significantly higher excess BMI loss and results were (MD = 3.92, 95% CI = [3.25, 4.59], p < 0.0001), and the results were homogenous (P=0.44). Similar results were found in the 24-month follow-up time, as RYBG showed a significantly higher excess BMI loss and results were (MD = 3.83, 95% CI = [2.89, 4.76], p < 0.0001). The total combined results of all the follow-up periods showed a significant superiority of RYGB when compared to OAGB and the results were (MD = 3.91, 95% CI = [3.37, 4.45], p <0.0001). The overall results were homogenous (overall p-value=0.82, I2=0%) (Figure 18).

Discussion

Our results revealed that OAGB was found to be more effective than RYGB in reducing BMI, while RYGB showed better results in overall weight and BMI loss. OAGB benefits included shorter operation and hospital stay times, along with fewer overall and endoscopy-related complications. On the other hand, RYGB was associated with higher rates of dyslipidemia remission and hypertension remission. No other outcomes showed a significant difference between the two techniques whether on long or short terms. Our systematic review and meta-analysis aim to provide updated insights for medical professionals, building upon the findings of Li's meta-analysis from 2022 [32]. Several factors motivated our study: the publication of numerous high-quality RCTs since the previous meta-analysis conducted in July 2022, and the retraction of one of the studies included in the prior analysis. These developments underscore the necessity for updated results to offer the most recent guidance to medical professionals.

Upper gastrointestinal endoscopy complications, which were associated with lower incidence in OAGB, may occur postoperatively directly or within two years. These complications include conditions such as hiatal hernia, bile or acid reflux, marginal ulcers, gastritis, and esophagitis [33]. There was no significant difference between RYGB and OAGB regarding intraoperative complications, such as minor liver injury, splenic capsule tear, and stapler misfire, as both techniques were found to have high safety and low incidence of intraoperative complications [34].

In the 2018 IFSO survey findings, OAGB ranked as the third most favored bariatric surgery option [35], contrasting with RYGB, which held the top position as the most frequently performed bariatric procedure from 2003 to 2013 [36], despite RYGB being more invasive and having higher recovery time compared to OAGB. Prior research has indicated that the learning curve for OAGB is shorter compared to RYGB, with implications that this curve is closely associated with surgical complications [37]. RYGB is considered a more complex procedure compared to OAGB due to technical challenges, particularly the high anastomosis near the esophagogastric junction. Surgeons typically opt for a retro-colic Roux-en-Y limb, especially in early experiences, to minimize tension on the mesentery [27].

Several previous studies have reported comparable weight loss and metabolic enhancements in both procedures equally [38, 39], including improvements in lipids, blood pressure, and type 2 diabetes remission, across both procedures which is similar to our findings [40]. However, OAGB has been associated with higher rates of nutritional issues, with severe malnutrition being more common after OAGB [40, 41]. Discrepancies in the length of bypassed intestine among studies may have contributed to variations in metabolic outcomes [41, 42]. To overcome these challenges we made a subgroup analysis for the data that was available in various follow-up periods.

Variable assessment methods were used to obtain results: Eskandaros et al. [21] used a unique scoring for symptoms. Patients experiencing symptoms of GERD completed a standardized questionnaire known as the Patient Assessment of Upper Gastrointestinal Disorder-Symptom Severity Index (PAGI-SYM). This questionnaire consists of 20 items divided into six categories, including all types of abdominal adverse events. Each item is scored on a 6-point Likert with ranges from 0 to 5. The scores from each category are averaged to obtain subscale scores, which are then averaged to calculate the final score. Patients with mild-to-moderate GERD symptoms (score between 0 and 2) underwent further evaluations including upper endoscopy, esophageal manometry, and 24-hour pH monitoring before surgery [43-45]. In addition, Fahmy et al. used a GERD score questionnaire to assess the GERD symptoms pre and post-operation and measure the improvement [22].

A previous study found that laparoscopic sleeve gastrectomy significantly impacted specific micronutrient deficiencies at six years post-surgery, highlighting the importance of oral supplementation for prevention and management [46]. There are no previously identified patient risk factors which were not validated as independent predictors, except for smoking [47]. Complications from bariatric surgery, such as malnutrition or rapid weight loss, may aggravate liver dysfunction in patients with pre-existing hepatic conditions, and in rare instances, this could precipitate the development of hepatic encephalopathy [48].

Our study possesses several notable strengths. Firstly, it exclusively comprises randomized controlled trials (RCTs) of moderate to high quality, ensuring robustness and reliability in our findings. We conducted subgroup analyses for all available outcomes across various follow-up periods, enhancing the depth and specificity of our results. By incorporating five new studies while excluding a retracted article from previous analyses, we mitigated bias and ensured the currency and accuracy of our data. Notably, our data demonstrated predominantly homogenous outcomes, with any heterogeneity effectively addressed through analytical techniques. Additionally, we addressed the issue of disparate follow-up durations by conducting subgroup analyses stratified by time, offering comprehensive insights for bariatric surgeons in diverse clinical scenarios.

However, our study also exhibits certain limitations. Some outcomes lacked sufficient data for subgroup analysis due to variability in follow-up durations among included studies. The absence of blinding procedures in certain studies may introduce bias, potentially influencing outcome assessments. Furthermore, variations in the assessment methods used to evaluate complications across studies could contribute to heterogeneity in our findings, warranting caution in their interpretation.

## Conclusions

The comparison of OAGB and RYGB in multiple studies reveals nuanced insights into their effectiveness and safety as bariatric surgery options. Both procedures show distinct advantages and challenges, highlighting the importance of personalized treatment plans for each patient according to the situation. Operation time and hospital stay outcomes favor OAGB, indicating it as a more efficient procedure with shorter recovery times, which is considered a preferable option for patients seeking a quicker return to daily life. In terms of surgical and early postoperative complications, the results are mixed. While there's no significant difference in intraoperative complications, early postoperative complications are significantly lower with OAGB. This suggests that OAGB might offer a safer postoperative profile in the immediate term. However, the significantly lower incidence of upper gastrointestinal endoscopy complications with RYGB could indicate a better long-term safety profile for this procedure. Follow-up outcomes on GERD symptoms, dumping syndrome, anemia, osteoarthritis remission, and dyslipidemia remission show no significant differences between the two techniques in most areas, suggesting that both procedures have similar long-term effects on these conditions. However, RYGB shows a significantly higher remission rate for hypertension and dyslipidemia, indicating potential benefits for patients with these comorbidities. RYGB demonstrates a significantly higher excess weight loss and excess BMI loss, suggesting that it may be more effective for substantial long-term weight reduction.
